# Inflammatory Properties of Diet and Glucose-Insulin Homeostasis in a Cohort of Iranian Adults

**DOI:** 10.3390/nu8110735

**Published:** 2016-11-18

**Authors:** Nazanin Moslehi, Behnaz Ehsani, Parvin Mirmiran, Nitin Shivappa, Maryam Tohidi, James R. Hébert, Fereidoun Azizi

**Affiliations:** 1 Nutrition and Endocrine Research Center, Research Institute for Endocrine Sciences, Shahid Beheshti University of Medical Sciences, Tehran 1985717413, Iran; moslehinazanin@yahoo.com or moslehinazanin@sbmu.ac.ir; 2 Department of Clinical Nutrition and Dietetics, Faculty of Nutrition Sciences and Food Technology, National Nutrition and Food Technology Research Institute, Shahid Beheshti University of Medical Sciences, Tehran 1981619573, Iran; behi.nutrist@gmail.com; 3 Cancer Prevention and Control Program, University of South Carolina, Columbia, SC 29208, USA; shivappa@mailbox.sc.edu (N.S.); Jhebert@mailbox.sc.edu (J.R.H.); 4 Department of Epidemiology and Biostatistics, Arnold School of Public Health, University of South Carolina, Columbia, SC 29208, USA; 5 Connecting Health Innovations, LLC, Columbia, SC 29201, USA; 6 Prevention of Metabolic Disorders Research Center, Research Institute for Endocrine Sciences, Shahid Beheshti University of Medical Sciences, Tehran 1985717413, Iran; tohidi@endocrine.ac.ir; 7 Endocrine Research Center, Research Institute for Endocrine Sciences, Shahid Beheshti University of Medical Sciences, Tehran 1985717413, Iran; azizi@endocrine.ac.ir

**Keywords:** dietary inflammatory index, glucose metabolism, insulin sensitivity, glucose tolerance abnormality

## Abstract

We aimed to investigate associations of the dietary inflammatory index (DII) with glucose-insulin homeostasis markers, and the risk of glucose intolerance. This cross-sectional study included 2975 adults from the Tehran Lipid and Glucose Study. Fasting plasma glucose (FPG), 2-h post-load glucose (2h-PG), and fasting serum insulin were measured. Homeostatic model assessment of insulin resistance index (HOMA-IR) and β-cell function (HOMA-B), and the quantitative insulin sensitivity check index (QUICKI) were calculated. Glucose tolerance abnormalities included impaired fasting glucose (IFG), impaired glucose tolerance (IGT), and type 2 diabetes (T2DM). DII scores were positively associated with 2h-PG (β = 0.04; *p* = 0.05). There was no significant linear trend across quartiles of DII for adjusted means of glucose-insulin homeostasis markers. Participants in the highest quartile of DII score tended to have higher FPG compared to those in the second quartile of DII score (5.46 vs. 5.38 mmol/L, *p* = 0.07) and higher fasting insulin and HOMA-IR compared to those in the lowest quartile (8.52 vs. 8.12 µU/mL for fasting insulin, *p* = 0.07; 2.06 vs. 1.96 for HOMA-IR, *p* = 0.08). No significant associations were observed between DII and risk of IFG, IGT, T2DM, and insulin resistance. Among glucose-insulin homeostasis markers, DII had a positive weak association only with 2h-PG.

## 1. Introduction

Type 2 Diabetes Mellitus (T2DM) is emerging as one of the most important epidemics of the 21st century, with devastating health and economic impacts [1]. It has been reported that 382 million people in the world had T2DM in 2013, a number expected to increase in the future, reaching 592 million in 2035 [2]. Globally, 1.5 million adult deaths and 89 million disability-adjusted life-years (DALY) in 2012 have been attributed to diabetes [3].

With the emergence of industrial agriculture and widespread food processing, there have been massive changes in the dietary behaviors and nutritional status of the world’s population. These changes constitute a significant contribution to the generation of T2DM and other inflammation-related diseases [4]. Observational studies from Iran indicate significant associations between dietary factors and markers of glucose metabolism, and risk of T2DM [5,6,7,8]. However, associations between quality aspects of diet, markers of glucose and insulin metabolism, and risk of glucose tolerance abnormalities such as impaired fasting glucose (IFG) and impaired glucose tolerance (IGT), and insulin resistance (IR) remain to be investigated.

Dietary components can exert pro-inflammatory or anti-inflammatory effects, which may influence the risk of inflammatory diseases [9,10]. The dietary inflammatory index (DII) is a newly developed tool for assessing the inflammatory properties of the diet based on intake of macronutrients, micronutrients and some other dietary constituents [11]. The DII score is calculated using inflammatory scores of each food parameters and their standardized intakes relative to global values. Previous studies have shown that DII is associated with increased inflammatory biomarkers [12,13], incidence of cardiovascular disease [14] and some cancers [15,16,17], lung function and asthma [18], adiposity measures [19], and bone mineral density in postmenopausal women [20]. Studies on the associations between DII and markers of glucose metabolism including fasting plasma glucose (FPG), 2-h post-load glucose (2h-PG), and insulin report inconsistent findings [21,22,23], and the associations between DII and the risk of glucose tolerance abnormalities including IFG, IGT, and T2DM have not been reported previously. Therefore, the present study aims to examine the associations of the DII with markers of glucose-insulin homeostasis, and with the risk of glucose tolerance abnormalities and insulin resistance in an Iranian population.

## 2. Methods

### 2.1. Participants

**Figure 1:**
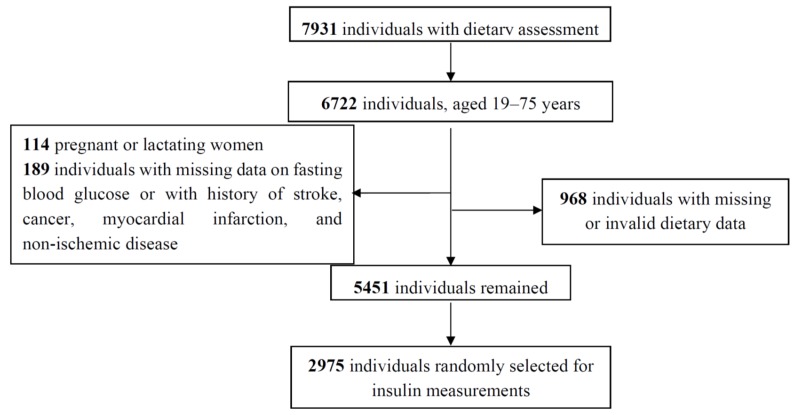
Selection of study participants.

This cross-sectional study is based on data from the Tehran Lipid and Glucose Study (TLGS), an ongoing community-based cohort study aiming to investigate the prevalence and incidence of chronic diseases and their risk factors [24]. The TLGS was initiated in 1999–2001 with 15,005 subjects, aged ≥ 3 years, who were residents of district 13 of Tehran. After determining the prevalence of chronic diseases and their risk factors at baseline examination, participants have been followed every three years to update their information. In this study, the data collected at the fourth follow-up examination (2009–2011) was used. Of 12,523 individuals, who participated in the fourth follow-up examination, 7931 individuals were randomly selected for dietary assessments. For the current study, 6722 men and women, aged 19–75 years were selected. Pregnant and lactating women and those with missing information on fasting blood glucose, those with a history of stroke, cancer, myocardial infarction and non-ischemic disease were excluded. Participants with total energy intake outside predefined limits (*n* = 645; total energy intake <800 kcal/day or >4200 kcal/day for men; <600 kcal/day or >3500 kcal/day for women) and individuals with outlier intakes of at least one of the food parameters (*n* = 323; ≥3.3 standard deviation (SD)) also were excluded. Finally, 5451 participants remained, of whom 2975 subjects were randomly selected for insulin measurements and further investigations in this study (Figure 1). The ethics committee of the Research Institute for Endocrine Sciences of Shahid Beheshti University of Medical Sciences approved the study protocol, and written informed consent was obtained from all participants.

### 2.2. Clinical and Biochemical Measurements

Information on demographic, past medical history, current medications use and smoking habits of participants were obtained using a pretested questionnaire administered by trained interviewers [24]. Subjects who smoked daily or occasionally were considered as current smokers; whereas non-smokers included those who had never smoked or those who had quit smoking. Weight and height were measured, and body mass index (BMI) was calculated. Blood pressure (BP) was measured by a standardized mercury sphygmomanometer, twice on the right arm in a seated position, after a rest period of 15 min; the average of the two measurements was considered as participant’s BP. Hypertension was defined as systolic blood pressure/diastolic blood pressure ≥130/85 or taking anti-hypertensive drugs. Physical activity levels were assessed using the Persian translated Modifiable Activity Questionnaire (MAQ) [25], which measured all three types of activity including leisure time, job and household activities in the past year and expressed as metabolic equivalent (MET) minutes per week. Physical activity was categorized into four groups (missing, and MET score in tertiles). Tertiles of physical activity were defined as ≤94.1, >94.1 to ≤498.5, and >498.5 MET-min/week.

Blood samples were taken from all participants at the TLGS research laboratory after an overnight fast of 12–14 h. FPG and 2-hPG were assayed by enzymatic colorimetric method using glucose oxidase (Pars Azmoon, Tehran, Iran) and a Selectra 2 Autoanalyzer (Vital scientific, Spankeren, The Netherlands) with both intra- and inter-assay coefficients of variation (CVs) less than 2%. Fasting serum insulin was determined by the electrochemiluminescence immunoassay (ECLIA) method, using Roche Diagnostics kits and the Roche/Hitachi Cobas e-411 analyzer (GmbH, Mannheim, Germany). Intra- and inter-assay CVs were 1.2% and 3.5%, respectively.

### 2.3. Outcome Definitions

Diabetes Mellitus was defined according to the American Diabetes Association (ADA), as fasting glucose ≥7.0 mmol/L or 2-hPG ≥11.1 mmol/L and/or use of anti-diabetic medication; IFG was defined as fasting glucose 5.6–6.9 mmol/L and IGT was defined as 2-hPG 7.8–11.0 mmol/L [26]. Insulin resistance/sensitivity were calculated as follows: Homeostatic model assessment insulin resistance index (HOMA-IR) = [fasting insulin (μU/mL) × fasting glucose (mmol/L)]/22.5; Homeostatic model of β-cell function (HOMA-B) = [20 × fasting insulin (μU/mL)]/fasting glucose (mmol/L) − 3.5; the quantitative insulin sensitivity check index (QUICKI) = 1/[log fasting insulin (μU/mL) + log fasting glucose (mg/dL)]. IR was defined as HOMA-IR ≥1.86 in women, and ≥1.34 in men [27].

### 2.4. Dietary Assessment and Calculation of the Dietary Inflammatory Index

Information about the usual diets of the participants during the preceding year was gathered using a validated and reliable semi-quantitative food frequency questionnaire (FFQ) [28,29], which consisted of 168 dietary items with standard portion sizes and participants were asked to designate their frequency of consumption for each food item, on a daily, weekly, monthly or yearly basis. These reported intakes were first converted to daily frequencies, and then were converted to grams. Energy and nutrient contents of food items were analyzed using the USDA Food Composition Table (FCT) and for traditional Iranian food that were not provided by the USDA FCT, the Iranian food composition table was used. Flavonoid contents of foods were calculated based on linkage with the USDA database for flavonoid content of selected foods.

Data on intakes of 37 food parameters including carbohydrate, protein, total fat, cholesterol, saturated fatty acids, trans fatty acids, monounsaturated fatty acids (MUFA), polyunsaturated fatty acids (PUFA), *n*-3 fatty acids, *n*-6 fatty acids, fiber, folic acid, niacin, riboflavin, thiamin, vitamin A, vitamin C, vitamin D, vitamin E, vitamin B6, vitamin B12, iron, zinc, selenium, magnesium, β-carotene, flavan-3-ol, flavones, flavonols, flavonones, anthocyanidins, isoflavones, onion, garlic, pepper, caffeine and tea were used to calculate the DII. All food parameters were adjusted for energy using the energy density method [30], therefore, energy-adjusted intakes of food parameters were used to construct of the DII. The DII construction procedure has been reported elsewhere [11]. Briefly, adjusted intake of food parameters for each individual is standardized to its corresponding global mean and standard deviation. The derived Z score values were converted to percentile and centered, by doubling the values and subtracting one, to normalize the scoring system and to avoid skewness. The centered percentile value for each food parameter is then multiplied by its respective overall food parameter score to obtain the food parameter-specific DII score. Finally, the DII score was determined by summing all of the food parameter-specific DII score. The greater the DII score, the more pro-inflammatory the diet and more negative scores demonstrate a more anti-inflammatory diet. Validity of the DII score was reported based on a 24-h dietary recall and a structured questionnaire similar to FFQ [13].

### 2.5. Statistical Analysis

Characteristics of participants across quartiles of the DII were compared using one-way analysis of variance (ANOVA) with Tukey’s post hoc test for continuous variables (presented as means ± SD) and chi-square tests for categorical variables (presented as percentage). All of the glucose metabolism markers were skewed; therefore, the natural logarithmic transformed (Ln) values were used in analyses. The associations between DII, as a continuous variable, and markers of glucose metabolism were assessed using linear regression analyses. Furthermore, adjusted geometric means of glucose metabolism markers across quartile of the DII score were determined using analysis of covariance (ANCOVA). To assess odds of glucose tolerance abnormalities and IR according to quartiles of DII score, the adjusted odds ratios (ORs) with 95% CIs were estimated using logistic regression analyses, considering the lowest quartile as the reference group. In all analyses, sex, age (continuous), smoking status (smoker/nonsmoker), physical activity (MET score in tertiles, or missing), a family history of diabetes (yes/no, or missing), hypertension (yes/no), glucose lowering medications (yes/no) and lipid lowering medications (yes/no) were included in model 1 as covariates. BMI (<18.5 kg/m^2^, 18.5–24.99 kg/m^2^, 25–29.99 kg/m^2^ and ≥30 kg/m^2^, or missing) was included in model 2 in addition to the covariates of model 1. In the models of assessing the risk of glucose tolerance abnormality and T2DM, we did not include glucose lowering medication since it was considered as criteria for diagnosis of T2DM. P for trend across quartiles was computed by assigning each participant the median score for the quartiles and modeling this value as a continuous variable. The Statistical Packages for Social Sciences (SPSS version 20; Chicago, IL, USA) were used to perform analyses and *p*-values ≤ 0.05 were considered significant.

## 3. Results

The mean (±SD) age of the study participants was 45 ± 11.7 years, and 56% of participants were women. The DII ranged from −5.82 to 5.23 with the median of −3.43 in the lowest quartile and 1.24 in the highest quartile. Diabetes risk factors and other characteristics of the participants according to the DII score quartiles are presented in Table 1. Participants in the highest quartile of DII score were significantly younger, more likely to be men, had the lowest BMI, and the lowest percentage of having hypertension and of using glucose- and lipid-lowering medications. Moreover, participants with a higher DII score were less physically active and had a higher proportion of smokers than those in the lowest quartile.

Higher DII, as a continuous variable, was weakly associated with higher 2h-PG after adjusting for sex, age, smoking status, physical activity, a family history of diabetes, hypertension, glucose lowering medications, lipid lowering medications, and the association remained significant after inclusion of BMI in the model. No significant associations were observed between DII and other markers of glucose-insulin homeostasis (Table 2).

Adjusted geometric means of glucose-insulin homeostasis markers within the quartiles of the DII are presented in Table 3. There was no significant linear trend across quartiles of DII for adjusted means of glucose-insulin homeostasis markers. However, participants in the highest quartile of DII score tended to have higher FPG compared to those in the second quartile of DII score (5.46 vs. 5.38 mmol/L for model 2, *p* = 0.07). After adjusting for all potential covariates (model 2) mean fasting insulin and HOMA-IR tended to be higher among those in the highest quartile of DII score than among those in the lowest quartile (8.52 vs. 8.12 µU/mL for fasting insulin *p* = 0.07; 2.06 vs. 1.96 for HOMA-IR, *p* = 0.08).

About 34% of the participants had a glucose tolerance abnormality, and 64% showed IR. Odds ratios for glucose intolerance outcomes based on the quartiles of DII are presented in Table 4. There were no significant associations between the DII and any individual glucose tolerance abnormalities. In addition, the risk of IR was not significantly different across quartiles of DII.

## 4. Discussion

In this study, the inflammatory properties of diets defined by DII in relation to glucose-insulin homeostasis markers and risk of glucose tolerance abnormalities were investigated in a cohort of Iranian adults. Higher DII was significantly associated with higher 2h-PG after adjustment for all covariates. No significant positive linear trend was observed between the DII score and geometric mean values of glucose-insulin homeostasis markers. No significant association was observed between DII and odds of glucose tolerance abnormalities and IR.

The DII score was developed to assess the overall inflammatory quality of diet by summing the individual inflammatory effects of different food parameters that have been published in the literature [11]. Three cross-sectional studies conducted in US and European populations investigated DII in relation to FBS, insulin, and HOMA-IR [21,22,23]. In a Dutch cohort study, each 1-SD increase in DII was associated with 0.9% higher FBS and 2.3% higher 2h-PG though these associations become non-significant after inclusion of BMI to the model [21]. In a study conducted in Luxembourg no association was observed between DII and FPG [23]. The risk of hyperglycemia defined as FPG ≥100 mg/dL or use of glucose lowering medication was increased gradually from quartile 1 to quartile 4 of DII, and the increased risk of individuals in quartile 4 of DII was significant, compared to those in quartile 1 after adjustment for age and BMI in police officer of US [22]. In contrast, in the Luxemburg population study DII was not significantly associated with the risk of hyperglycemia [23]. To best of our knowledge, the associations between DII and glucose tolerance abnormalities including IFG, IGT, T2DM, and IR have not been previously examined.

Findings of our study suggest that the DII is not associated with fasting insulin secretion, β-cell function, and sensitivity to insulin. Therefore, the slightly positive association between the DII score and 2h-PG may imply the role of DII in regulation of postprandial glucose through non-hepatic mechanisms. In addition, the observation of significant association between DII and 2h-PG may be through the effects on postprandial insulin, which has not been measured in this study. To confirm the latter mechanism, investigating the associations between DII and postprandial markers of insulin are required in future studies. However, the magnitude of the association between the DII and 2h-PG was not sufficient enough to affect the risk of IGT in this study. The Dutch cohort study reported a 3.5% increase in HOMA-IR per 1-SD increase in DII in a crude model and after adjusting for age, sex, physical activity, smoking, family history of diabetes, lipid and glucose medication, an association that became non-significant by adding BMI to the model [21]. However, in the Luxembourg study population no significant association was found between DII, insulin and HOMA-IR [23].

Standardizing the intake of each food parameter based on world mean and SD render the DII scores comparable across any population. Ideally, 45 food parameters are suggested for use to calculate the DII score [11]. However, the number of food parameters varies widely between studies, which complicate comparability of the dietary inflammatory properties of diet estimated using different dietary assessment instruments in different populations. Variation in DII across populations and differences in the number and type of food parameter used to calculate DII might result in inconsistent findings. The majority participants in our study population had anti-inflammatory diets, and the mean score of DII in our study was −1.13 ± 1.92, which was lower than those were reported in US populations [13,22]. Differences in DII scores in our study participants might not be large enough to allow for detecting associations with the risk of glucose intolerance abnormalities. Further studies are needed to examine whether DII scores are associated with glucose-insulin homeostasis and risk of glucose tolerance abnormalities.

Objectively identified cases of diabetes, using Iranian cutoff values to identify insulin resistance, using validated questionnaires of assessing dietary intakes and physical activity in our study population, examining the association between DII with HOMA-B, a marker of β-cell function in addition to markers of glucose metabolism and insulin action are some strengths of our study. However, its cross-sectional design is the main limitation. In addition, despite using a validated FFQ, self-reporting of diet is subject to bias and misreporting. Lastly, our participants are not a representative sample of Iranian populations, and present findings cannot be generalized to all Iranian populations.

## 5. Conclusions

In conclusion, among glucose-insulin homeostasis markers, DII had a positive weak association only with 2h-PG. DII was not significantly associated with risk of glucose tolerance abnormalities and IR. Our findings could not show the associations of inflammation induced by diet with the risk of glucose intolerance disorders.

## Figures and Tables

**Table 1:** Characteristics of participants according to the quartiles of the dietary inflammatory index (DII).

Characteristics	Quartiles of the DII				
	Q1 (*n* = 743)	Q2 (*n* = 744)	Q3 (*n* = 744)	Q4 (*n* = 744)	*p* *
DII					
Median	−3.43	−1.96	−0.48	1.24	
Minimum, maximum	−5.82, <−2.67	−2.67, <−1.23	−1.23, <0.29	0.29, 5.23	
Age, years	47.8 ± 11.6	45.8 ± 11.9	43.5 ± 11.3	41.9 ± 11.3	<0.001
Men	209 (16.1)	292 (22.4)	375 (28.8)	428 (32.8)	<0.001
Smoking	27 (11.9)	47 (20.7)	73 (32.2)	80 (35.2)	<0.001
Body mass index ^†^, kg/m^2^	28.8 ± 4.63	28.0 ± 4.41	27.8 ± 4.47	27.4 ± 4.41	<0.001
Physical activity ^‡^, MET-min/week	652 ± 893	561 ± 768	505 ± 762	469 ± 780	<0.001
Family history of diabetes ^§^	76 (23.5)	85 (26.3)	80 (24.8)	82 (25.4)	0.90
Glucose-lowering medication	62 (42.8)	36 (24.8)	24 (16.6)	23 (15.9)	<0.001
Lipid-lowering medication	74 (36.5)	52 (25.6)	44 (21.7)	33 (16.3)	<0.001
Hypertension ^¶^	152 (31.5)	127 (26.3)	105 (21.8)	98 (20.3)	0.001

Data are presented as mean ± standard deviation or n (%). * Chi-square test was used for categorical variables; ANOVA was used for continuous variables; ^†^ Missing for 15 subjects; ^‡^ Missing for 296 subjects; ^§^ Missing for 2 subjects; ^¶^ Hypertension: SBP/DBP ≥ 130/85 or use of hypertensive medication.

**Table 2:** Beta coefficients of the association between the dietary inflammatory index (DII) and glucose-insulin homeostasis markers *.

Variables	Model 1 ^†^		Model 2 ^‡^	
	Standardized Beta	*p*	Standardized Beta	*p*
Fasting plasma glucose, mmol/L	0.008	0.61	0.01	0.52
Postload glucose, mmol/L	0.03	0.05	0.04	0.05
Fasting insulin, μU/mL	0.01	0.42	0.02	0.17
HOMA-IR	0.02	0.38	0.02	0.15
HOMA-B	0.007	0.71	0.01	0.48
QUICKI	−0.01	0.43	−0.02	0.18

* Values were loge transformed for the analysis. ^†^ Adjusted for sex, age (continuous), smoking status (smoker, nonsmoker), physical activity (low, medium, high, or missing), a family history of diabetes (yes, no, or missing), hypertension (yes, no), glucose lowering medications (yes, no), lipid lowering medications (yes, no); ^‡^ Further adjusted for variables in model 1 plus BMI category (in kg/m^2^; <18.5, 18.5–24.99, 25.0–29.99, ≥30.0, or missing).

**Table 3:** Adjusted mean (95% confidence interval) of glucose-insulin homeostasis markers according to quartiles of the dietary inflammatory index (DII).

Variables	Quartiles of the Dietary Inflammatory Index										
	Q1 (*n* = 743)		Q2 (*n* = 744)		Q3 (*n* = 744)		Q4 (*n* = 744)		*p* 1 vs. 4	*p* 2 vs. 4	*p*_trend_
	Geometric Means	95% CI	Geometric Means	95% CI	Geometric Means	95% CI	Geometric Means	95% CI			
**Fasting plasma glucose, mmol/L**											
Model 1	5.45	5.39, 5.52	5.38	5.32, 5.44	5.46	5.39, 5.52	5.46	5.39, 5.52	0.89	0.08	0.23
Model 2	5.45	5.39, 5.51	5.38	5.32, 5.44	5.46	5.39, 5.51	5.46	5.40, 5.52	0.82	0.07	0.24
**Postload glucose, mmol/L**											
Model 1	5.67	5.51, 5.78	5.72	5.61, 5.83	5.83	5.67, 5.94	5.78	5.67, 5.89	0.15	0.53	0.28
Model 2	5.67	5.51, 5.78	5.72	5.61, 5.83	5.83	5.67, 5.94	5.78	5.67, 5.89	0.13	0.51	0.27
**Fasting insulin, μU/mL**											
Model 1	8.15	7.84, 8.47	8.26	7.95, 8.57	8.23	7.93, 8.55	8.48	8.16, 8.81	0.16	0.33	0.53
Model 2	8.12	7.83, 8.41	8.26	7.99, 8.56	8.21	7.94, 8.50	8.52	8.22, 8.82	0.07	0.24	0.29
**HOMA-IR**											
Model 1	1.97	1.89, 2.06	1.97	1.89, 2.05	2.00	1.91, 2.08	2.05	1.97, 2.14	0.18	0.17	0.49
Model 2	1.96	1.89, 2.04	1.98	1.90, 2.05	1.99	1.91, 2.06	2.06	1.99, 2.14	0.08	0.11	0.28
**HOMA-B**											
Model 1	89.7	86.0, 93.5	93.4	89.5, 97.2	90.6	86.9, 94.4	92.9	89.0, 96.8	0.26	0.88	0.48
Model 2	89.4	85.8, 93.1	93.4	89.8, 97.2	90.5	86.9, 94.1	93.1	89.5, 97.0	0.17	0.92	0.34
**QUICKI**											
Model 1	0.35	0.34, 0.35	0.35	0.34, 0.35	0.34	0.34, 0.35	0.34	0.34, 0.35	0.20	0.22	0.55
Model 2	0.35	0.34, 0.35	0.35	0.34, 0.35	0.34	0.34, 0.35	0.34	0.34, 0.35	0.09	0.16	0.33

Model 1 adjusted for sex, age (continuous), smoking status (smoker, nonsmoker), physical activity (low, medium high, or missing), a family history of diabetes (yes, no, or missing), hypertension (yes, no), glucose lowering medications (yes, no) and lipid lowering medications (yes, no). Model 2 adjusted for variables in model 1 plus BMI (in kg/m^2^; <18.5, 18.5–24.99, 25.0–29.99, ≥30.0, or missing).

**Table 4:** Odds ratios (95% CI) for glucose intolerance outcomes according to the quartiles of the dietary inflammatory Index (DII).

Variables	Dietary Inflammatory Index							
	Q1	Q2		Q3		Q4		*p*_trend_
		OR	95% CI	OR	95% CI	OR	95% CI	
**Glucose tolerance abnormality** * (*n* = 1007)								
Model 1	1.00	0.94	0.74, 1.19	1.11	0.87, 1.41	1.15	0.90, 1.47	0.14
Model 2	1.00	0.94	0.74, 1.19	1.10	0.87, 1.40	1.15	0.90, 1.48	0.14
**Impaired fasting glucose** ^†^ (*n* = 590)								
Model 1	1.00	0.96	0.74, 1.25	1.11	0.85, 1.44	1.09	0.83, 1.44	0.36
Model 2	1.00	0.97	0.74, 1.26	1.10	0.84, 1.43	1.09	0.83, 1.44	0.37
**Impaired glucose tolerance** ^‡^ (*n* = 259)								
Model 1	1.00	0.96	0.66, 1.39	0.99	0.68, 1.46	1.24	0.85, 1.81	0.25
Model 2	1.00	0.96	0.66, 1.40	0.98	0.67, 1.45	1.24	0.84, 1.81	0.27
**Type 2 diabetes** ^§^ (*n* = 286)								
Model 1	1.00	0.96	0.67, 1.38	1.06	0.73, 1.54	0.99	0.66, 1.47	0.93
Model 2	1.00	0.96	0.67, 1.38	1.05	0.72, 1.53	0.98	0.66, 1.47	0.94
**Insulin resistance** ^||^ (*n* = 1923)								
Model 1 ^¶^	1.00	1.01	0.81, 1.26	0.97	0.77, 1.22	1.11	0.87, 1.40	0.47
Model 2	1.00	1.05	0.83, 1.32	0.98	0.77, 1.25	1.18	0.91, 1.51	0.29

Model 1 adjusted for age (continuous), smoking status (smoker, nonsmoker), physical activity (low, medium, high, or missing), a family history of diabetes (yes, no), hypertension (yes, no), lipid lowering medications (yes, no). Model 2 adjusted for variables in model 1 plus BMI (<18.5, 18.5–24.99, 25.0–29.99, ≥30.0 kg/m^2^, or missing). * Including impaired fasting glucose, impaired glucose tolerance, and type 2 diabetes; ^†^ Impaired fasting glucose was defined as fasting glucose 100–125 mg/dL; ^‡^ Impaired glucose tolerance was defined as 2-h postload glucose 140–199 mg/dL; ^§^ Diabetes Mellitus was defined as fasting glucose ≥126 mg/dL or 2-h glucose ≥200 mg/dL and/or use of anti-diabetic medication; ^||^ Insulin resistance was defined as HOMA-IR ≥ 1.86 in women and ≥1.34 in men; ^¶^ adjusted for glucose lowering medication.
